# From Human Geography to Biological Invasions: The Black Rat Distribution in the Changing Southeastern of Senegal

**DOI:** 10.1371/journal.pone.0163547

**Published:** 2016-09-23

**Authors:** Héloïse Lucaccioni, Laurent Granjon, Ambroise Dalecky, Odile Fossati, Jean Le Fur, Jean-Marc Duplantier, Pascal Handschumacher

**Affiliations:** 1 Université Paris Ouest Nanterre La Défense, UMR 7533 LADYSS, Nanterre, France; 2 Institut Français de Recherche en Afrique, UMIFRE CNRS 24, Ibadan, Nigeria; 3 IRD, UMR CBGP, Montferrier-sur-Lez, France; 4 IRD, Aix Marseille Univ, LPED, Marseille, France; 5 IRD, Aix Marseille Univ, INSERM, IRD, SESSTIM, Sciences Economiques & Sociales de la Santé & Traitement de l’Information Médicale, Marseille, France; São Paulo State University, BRAZIL

## Abstract

In the contemporary context of zoonosis emergence and spread, invasive species are a major issue since they represent potential pathogen hosts. Even though many progresses have been done to understand and predict spatial patterns of invasive species, the challenge to identify the underlying determinants of their distribution remains a central question in invasion biology. This is particularly exacerbated in the case of commensal species that strictly depend on humankind for dispersal and perennial establishment of new populations. The distribution of these species is predicted to be influenced by dispersal opportunities and conditions acting on establishment and proliferation, such as environmental characteristics, including spatio-temporal components of the human societies. We propose to contribute to the understanding of the recent spread of a major invasive rodent species, the black rat (*Rattus rattus)*, in the changing southeastern of Senegal. We address the factors that promote the dispersal and distribution of this invasive rodent from the perspective of human geography. We first describe characteristics of human settlements in terms of social and spatial organization of human societies (i.e. economic activities, commercial and agricultural networks, roads connectivity). We then explore the relationship between these characteristics and the distribution of this invasive rodent. Finally we propose that historical and contemporary dynamics of human societies have contributed to the risk of invasion of the black rat. We argue that the diffusion processes of invasive species cannot be considered as a result of the spatial structure only (i.e. connectivity and distance), but as a part of the human territory that includes the social and spatial organization. Results suggest that the distribution of invasive rodents partly results from the contemporary and inherited human socio-spatial systems, beyond the existence of suitable ecological conditions that are classically investigated by biologists.

## Introduction

Biological invasions are recognized to be promoted by human-induced global change [1], including increased trade and transportation [2]. In the meantime, worldwide concern has raised about zoonosis emergence and spread [3] linked with the global establishment of pathogens housed by invasive hosts [4,5]. In this context, invasive rodents, including the black rat (*Rattus rattus*) [6,7] are of special concern because they are widespread and known hosts of many pathogens [8,9].

The detection and prediction of the spatial dynamics of such invasive processes is a challenge [5]. Bioecological or spatial modeling approaches have been explored [10–13], but privileging a global scale and ending up with potential rather than realized distribution area of the host species. The dispersal and distribution of invasive species reflects a spatial process of diffusion, i.e. the spread of species across space and time [14]. Furthermore, the process of invasion refers to different stages: introduction, establishment, and proliferation. One consequence is that the factors of spatial diffusion, such as transportation, are not sufficient to predict the success of the invasion. We propose a paradigm shift by claiming that the single concept of spatial diffusion covers in fact two realities: the first constitutes the connectivity of places or spaces through networks, which act as support for introduction of the invasive species; the second covers the production by human societies of favorable places or spaces, including habitat suitability for establishment and proliferation. These two realities interact with one another such that the same links do not induce the same effects and dynamics according to places, while same places evolve according to the nature of the links to which they are connected. This limits the scope of the diffusion approach by causes and consequences analysis, rather than by a global understanding of interactions in socio-spatial systems at different scales. Hence, we argue that space is not just a physical neutral support of spatial processes, but human societies shape heterogeneous places, spaces, and spatial relationships among them [15]. Thus they participate in the risk of expansion of invasive species (e.g. rodents) beyond the existence of suitable biotopes classically investigated by ecologists. In this context we explore the recent expansion of the black rat in Southeastern Senegal by integrating human geography and ecology. Such an approach implies to collect multi-disciplinary data about human territories under transformations and rodents distribution.

In Senegal, the compilation of historical archives and recent trapping data together with genetic investigations has permitted to trace back the black rat invasion [16]. The species was first introduced in major ports in the 15^th^-16^th^ centuries. From there, it has spread in the country through colonial trade ways, first fluvial, then railway, and last tarred roads. In the 1990’s the black rat occupied the southern third of Senegal, and The Gambia, to the except of the extreme south east (Kedougou region). It only reached and maintained a permanent population in the Kedougou town after the asphalted road connected it with the regional capital town of Southeastern Senegal, Tambacounda, in 1998 [16].

In the last two decades Southeastern Senegal has known a progressive opening following the development of road networks, and the development of international trade and mining activities [17,18]. The risks of commensal rodent invasion in this increased traffic context are serious, since commensal rodents depend on human transportation to spread.

In order to understand how socio-spatial systems built by human societies may promote the invasion of the black rat in Southeastern Senegal among suitable biotopes: 1) we assess the socio-spatial heterogeneity of the invaded locations (e.g., economical activities, transportation, trade and agricultural networks), 2) we analyze the spatial patterns of the black rat occurrence and abundance and their relation to the mentioned socio-spatial heterogeneity, 3) we evidence socio-spatial systems and the social processes that shape them through the understanding of historical and contemporary dynamics of this human territory. Thus, we provide an original perspective on biological invasions complementary to studies that explore the occurrence and abundance of invasive rodents according to ecological conditions only [19].

We propose that the black rat invasion could be understood as the cross-combined effect of socio-spatial and ecological systems. Overall, our findings concur with previous studies on the importance of busy locations (hubs) as well as seemingly unimportant transport nodes [20]. Notwithstanding we introduce important nuances to this paradigm by showing that “hubs” definition cannot be reduced to spatial structures of a transportation network only, but also the social and spatial relationships among these structures have to be taken into account. Hence we emphasize the importance of understanding the invasion through a multidisciplinary framework, e.g. human geography and animal ecology. Such an approach can provide keys to appraise, and possibly manage zoonosis diffusion risk associated with rodent hosts.

## Material and Methods

### Study Area and Sampling

**Fig 1:**
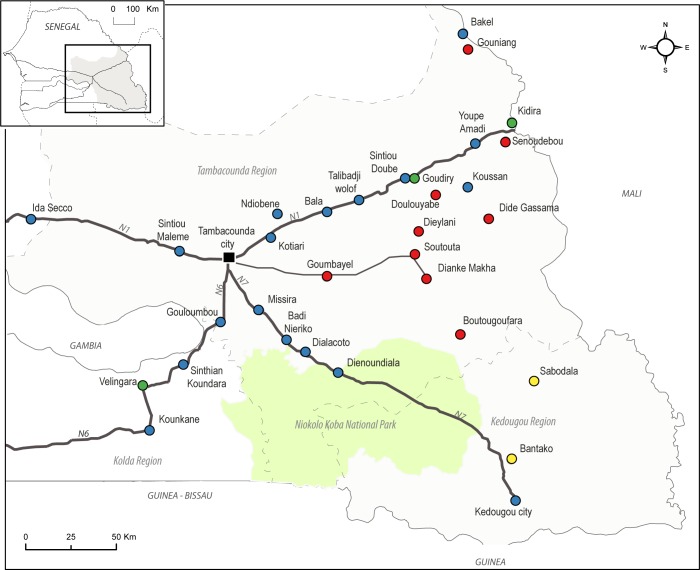
Study area and survey sites. **Clusters from the “synthetic socio-spatial” typology.** Study area and survey sites; black bold lines = national tarred roads; black thin lines = main untarred road; dashed grey lines = regional boundaries; the Dakar-Mali railway is strictly parallel to N1 road; Clusters from the "synthetic socio-spatial" typology (blue dots: rural localities with structuring capacities; red dots: marginal localities; green dots: urban-like localities; yellow dots: gold mining villages); Localities with socio-spatial data only: Gouniang, Dialacoto, Dienoundiala, Missira, Senoudebou.

A total of 32 localities were prospected, based on previous knowledge on the black rat distribution [21] in Southeastern Senegal (Fig 1).

### Rodent Data Collection

Small mammals were caught in single capture live traps between May 2012 and April 2015, indoors in 27 human settlements (villages or towns; hereafter referenced as localities), as *R*. *rattus* is strictly commensal (i.e. confined to human buildings) in Sahelo-Sudanian West Africa [21]. We used both Sherman folding box traps (8 × 9 × 23 cm; H.B. Sherman Traps, Inc., Tallahassee, Florida, USA) and wire-mesh live traps (8.5 × 8.5 × 26.5 cm; locally made). Typically two traps were set per room inside buildings (dwelling houses, storehouses or shops): one wire-mesh and one Sherman (occasionally four traps in large storage rooms), for trapping sessions of one to four consecutive nights. Hereafter only the two first nights of trapping were considered, which represent 98.3% of total sampling effort and 99.6% of captures of *R*. *rattus* individuals. For all sampled buildings, we recorded the geographical coordinates with a GPS device (Garmin Colorado 400, Olathe, USA). A trapping station was defined as the physical place where a single trap was set during a given trapping session. A variable number of traps was used in each locality with a median sampling effort of 218 trap nights per locality (min. = 131, max. = 876). Traps, baited with peanut butter supplemented with fresh onions, were set in the afternoon and checked in the morning. Animals were treated in a human manner, in accordance with requirements of the Senegalese legislation and following the guidelines of the American Society of Mammalogists [22]. The small mammals captured were euthanatized by cervical dislocation, as recommended by Mills et al. (1995) [23]. Small mammal species nomenclature follows Wilson and Reeder (2005) [24]. Most of the trapped individuals could be identified to the species level based on morphological, ecological and biogeographical knowledge of the rodent fauna of Sahelo-Soudanian Africa [21]. Captures of rodents excluded national parks and protected areas, did not involve endangered or protected species (CITES, IUCN, and national guidelines), and were conducted as part of research agreements between IRD, national institutions, national ministries of health and of scientific research. Prior informed consent to enter and conduct the study in human settlements was given by the appropriate institutional, traditional and familial local authorities. Trapping activities were approved by the Senegalese Ethics Committee, on behalf of the Pasteur Institute of Dakar, as part of the ChanCiRa research program.

The probability of presence of *R*. *rattus* at a trapping station was estimated within the framework of occupancy modeling, accounting for imperfect detection and for the potential effects of covariables (see below).

Whether *R*. *rattus* was the numerical dominant captured small mammal species (even if it does not exceed 50% of the captures) was estimated at the locality scale as a binary variable (Yes/No).

### Human Geography Data

Socio-spatial data were collected between April and June 2013 in 32 localities, including the 27 where small mammals were trapped. Detailed interviews with appropriate representatives (village headman, mayor, president of the administrative “Rural Community”, locality notables) and summary interviews with relevant stakeholders (chief of agricultural warehouses/cereal banks, shopkeepers, head of garage) were realized to obtain 19 categorical variables describing three topics: economic activities; supply storage and trade; structural links (Table 1). This dataset was complemented with archive data from both quantitative and qualitative sources like statistical or spatial data, scientific and NGO's articles, grey literature.

**Table 1:** Input categorical variables for MCA per topic.

Activities	Supply storage and trade	Structural links
Main economic activity practiced in the locality	Number of shops per capita	Untarred or tarred road
Secondary economic activity practiced in the locality	Type of markets in the locality (permanent, weekly, both, none)	Distance to tarred road
Practice of gardening	Distance to weekly market	Frequency of transportation line
Practice of trade	Type of collective storage of agricultural products*	Distance to Tambacounda (northern regional capital)
Exploitation of natural resources (e.g., charcoal or woodcutting, gathering, hunting, fishing)		Existence of direct transportation line toTambacounda
Practice of artisanal gold mining		Existence of direct transportation line to Kedougou (southeastern regional capital)
		Existence of direct transportation line to Kidira (secondary north town, rail and road border with Mali)
		Existence of direct transportation line to Goudiry (secondary north town, and departmental capital)
		Existence of direct transportation line to Kolda (southwestern regional capital)

Note: The variables are qualitative (categorical) either by nature or after being categorized from an estimation of quantities given by the interlocutors.

*Cereal banks or similar community-run storehouses, agricultural warehouses of inputs (seeds, implements) for the administrative “Rural Community”, inputs and collection centers structuring the intervention areas of agro-industrial exploitation of cotton by the Société de Développement et des Fibres Textiles (SODEFITEX).

### Data Analysis

#### Assessing Socio-Spatial Heterogeneities of the Localities

On each topic, Multiple Correspondence Analysis (MCA) was performed on the socio-spatial categorical variables that describe the 32 localities (Table 1). This data analysis technique is an extension of Correspondence Analysis which allows application to categorical dependent variables. The output includes factors scores and a graphical display of the modalities and localities interpreted based on the proximity between points in low-dimensional scatterplots [25,26]. MCA was here used as a pre-processing step before clustering method [27]. We then performed Hierarchical Cluster Analysis (HCA) on the first principal components of the previous MCA as continuous input variables [27]. This clustering method builds groups of localities by minimizing infra-cluster distance and maximizing inter-cluster distance using the Ward’s minimum variance criterion [28].

The combination of the three typologies obtained from each topic was finally used to build a “synthetic socio-spatial” typology by applying the same process of MCA and HCA [29]. The obtained synthetic typology forms a new categorical variable that describes the 32 localities according to their cluster.

#### Testing the Variability of the Rodent Distribution among the Socio-Spatial Typologies

In order to determine whether there are any differences between the rodent data and the synthetic socio-spatial typology, two analyses were performed on the 27 localities with rodent data.

The association between the binary rodent variable “numerical dominance of *R*. *rattus*” and the categorical socio-spatial clusters derived from the synthetic typology was tested with a two-tailed Fisher’s exact test of independence using the localities as observational units.

The covariation between the occurrence of the black rat and the synthetic socio-spatial characteristics of the localities was further tested using the framework of occupancy modeling accounting for imperfect detection [30,31] using the trapping stations as observational units and the two first nights of trapping as temporal replicates [19,32]. We built a logistic regression model in which the explanatory variable is the value assigned to each trapping station from the coordinates of its locality on each of the two first principal components of the synthetic MCA. Analyses were performed using the software PRESENCE 10.9 [33] for model selection following the Akaike criterion and for estimating the parameters of the models in Maximum-Likelihood.

#### Evidencing Socio-Spatial Systems Favorable to the Black Rat Invasion

The last step requires a qualitative approach typically used in human geography [34]. It consists in “making sense” of the previously evidenced spatial heterogeneities of the invaded localities, and of the results of the statistical analyses of covariation between *R*. *rattus* presence and locality characteristics described above. We confront socio-spatial characteristics and rodent data with their ecological, social, and economical context. This step allow us to move from patterns to processes by highlighting socio-spatial systems and the historical and contemporary social processes that have produced them.

## Results

### Socio-Spatial Typologies of the Localities

In the MCA on the “Activities” topic, the first two components represented respectively 67.14% and 28.43% of the variance. They were interpreted as the opposition between commercial localities with diversified economic activities and agricultural localities (axis 1), and the importance of gold mining (axis 2). Four clusters were then obtained from the HCA according to the weight of agriculture or trade, and degree of diversification or specialization of economic activities (Table 2A).

**Table 2:** Description of clusters from the socio-spatial typologies per topic.

**a—Activities:** *Importance of agriculture or trade and degree of diversification of economic activities*				
**Commercial localities (n = 3)**	**Gold mining villages (n = 2)**		**Poorly diversified agricultural localities (n = 9)**	**Highly diversified agricultural localities (n = 18)**
Agriculture as secondary activity; High diversification of activities	Important commercial activity; No exploitation of natural resources		Complementary activities oriented toward exploitation of natural resources	Importance of trade activities
**b—Supply storage and trade:** *Importance of trade*, *supply storage*, *and access to markets*				
**Major commercial centers (n = 5)**	**Localities with structuring role in trade and supply storage activities (n = 7)**		**Localities variably integrated to trade activities and developing agricultural storage strategies (n = 4)**	**Localities on the margins of trade activities with a tendency to self-subsistence (n = 16)**
Main administrative centers; Permanent markets; No cereal banks, mainly no collective supply storage of agricultural products; High number of shops per capita	Good access to permanent and weekly markets; Mainly ensuring supply storage responsibility of agricultural inputs for the “Rural Communities”, or input and collection centers for cotton cash crops; Relatively low number of shops per capita but structuring role as exchange places and supply storage centers		Agricultural production and exchange places; Variable access to weekly markets; Mainly ensuring supply storage for the “Rural Communities”; Variable use of cereal banks; Medium number of shops per capita	No market and at variable distance to weekly ones; Predominantly collective storage of agricultural products, in particular cereal banks; Diversity of supply storage types and a non negligible part without any; Mainly low number of shops per capita
**c—Structural links:** *Degree of integration to transportation network*, *orientation towards main or secondary*, *and “northern” or “southern” towns*				
**Well connected northern localities (n = 9)**	**Well connected southern localities (n = 10)**	**Bush villages with efficient connections (n = 4)**	**Bush villages with moderate connections (n = 5)**	**Weakly connected localities (n = 4)**
Along tarred road; well connected to major and secondary “northern” towns (Tambacounda, Goudiry, Kidira); High frequency of transportation line	Along tarred road, preferentially oriented toward Tambacounda and “southern” towns (Kedougou, Kolda)	Far from tarred road; High frequency of transportation line; Direct connection to major and secondary “northern” towns (Tambacounda, Goudiry)	Far from tarred road; Mainly directed toward secondary towns only	Mainly far away from tarred road; No transportation line; Random connections to secondary towns only

The first three components of the MCA on the “Supply storage and trade” captured respectively 50.87%, 33.72%, and 12.65% of the variance. They described the degree of structuring capacities in terms of supply storage (axis 1), the opposition between places of trade and places of agricultural production (axis 2), and strategies in terms of supply and storage management (axes 3). The typology then provided four clusters distinguished by trade weight, importance of supply storage, and access to permanent or weekly markets (Table 2B).

The MCA performed on the “Structural links” topic showed the first two components to represent 67% and 25% of the variance, respectively. They were interpreted as a gradient of connectivity to road and transportation networks (axis 1), and related to the position and orientation on the road and transportation networks (axis 2). The typology distinguished five clusters depending on the degree of connectivity with the transportation network and the orientation of transportation lines towards main/secondary, and “northern”/“southern” towns (Table 2C).

**Fig 2:**
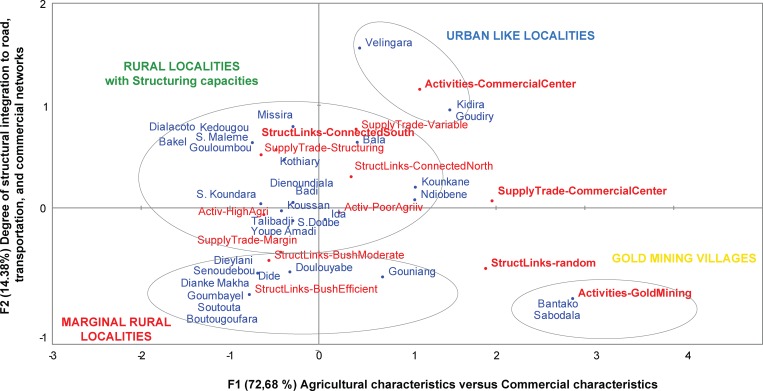
Asymetric biplot of the first two components for the synthetic socio-spatial MCA. Red dots: modalities of the input variables (from the previous thematic typologies); blue dots: localities (observations); grey circle = clusters from the "synthetic socio-spatial" typology below.

In the “synthetic socio-spatial” MCA realized on the combination of the three previous typologies, the first two components bore 72.68% and 14.8% of the variance, respectively. The first component was interpreted as the opposition between commercial and agricultural characteristics. On the positive side of this axis we observed the contribution of modalities that reflect a typical commercial profile such as in gold mining villages and commercial localities (as identified through the “Activities” typology). The negative side of this axis was associated with modalities that reflect a typical agricultural profile such as in highly diversified agricultural localities (as derived from the “Activities” typology), and localities with structuring role in trade and supply storage activities (as seen from the “Supply storage and Trade Activities”). The second component was interpreted as the degree of structural integration to road, transportation, and commercial networks. The positive side of this second axis represented modalities that figure a high degree of connectivity to networks through commercial localities (as in the “Activities” typology), and well connected southern localities (as from the “Structural links” typology). On its negative side, we found the contribution of modalities that represent a low degree of connectivity through weakly connected localities (from the “Structural links” typology), and localities on the margins of trade activities with a tendency to self-subsistence (as evidenced in the « Supply storage and trade” typology). (Fig 2)

The HCA performed on the locality coordinates on these first two principal components led to the “synthetic socio-spatial” typology partitioned into four clusters (Fig 1). These are defined according to their degree of trade activities, socio-territorial structuring capacities, and connection to transportation networks:

- Localities specialized in gold mining (n = 2) appear as commercial localities devoted to gold mining to the detriment of agriculture. They do not possess any agricultural supply storage and they appear weakly integrated to transportation networks;
- Urban-like localities (n = 3) ensure a structuring role in the human territory as major commercial and transportation nodes;
- Rural localities with structuring capacities (n = 18) have a predominantly agricultural profile with diversified activities and are well connected to tarred road and toward main towns. Moreover they are well integrated to trade and market activities and are of major importance for agricultural supply storage.
- Marginal rural localities (n = 9) mainly rely on subsidence agricultural activities and so practice supply storage, especially in the form of community-run storehouses such as cereal banks. They are variably integrated to transportation networks.

### Trapping Results

Trapping effort was of 6831 trap nights for a total of 5169 trapping stations (2610 equipped with a wire-mesh trap and 2559 with a Sherman box). In total, we captured 1486 small mammal individuals including 470 *R*. *rattus* individuals. Other commensal captured taxa were: *Crocidura* cf. *olivieri*, *Mus musculus domesticu*s, *Mastomys natalensis*, *Mastomys erythroleucus*, *Arvicanthis niloticus* and *Praomys daltoni*.

### Numerical Dominance of *R*. *rattus* in Localities

In the “synthetic socio-spatial” typology, *R*. *rattus* is the most abundant species in none of the urban-like localities or gold mining villages, in only four of the 15 (26.7%) rural localities with structuring capacities, but in six of the seven (85.7%) marginal rural localities (p = 0.012). The Fisher’s exact test performed between the variable “numerical dominance of *R*. *rattus*” and the socio-spatial clusters was significant.

### Probability of Occupancy of *R*. *rattus* at the Trapping Stations

The probability of detection of *R*. *rattus* in a trapping night given the species was present, as calculated through occupancy modeling, varied with the model of trap and decreased between the first and the second night of trapping (Sherman traps: 0.38 [95% confidence interval: 0.30–0.47] and 0.23 [0.17–0.32] for the first and second night, respectively; wire-mesh traps: 0.61 [0.49–0.71] and 0.43 [0.33–0.54] for the first and second night, respectively).

**Fig 3:**
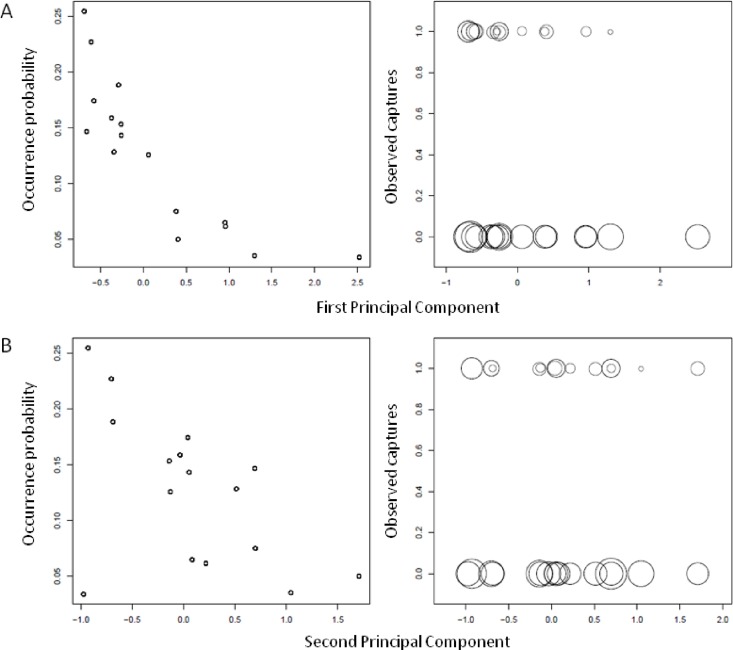
**Individual site estimates of the occurrence probability of *R*. *rattus* according to data of human geography in 27 localities in southeastern Senegal (left), and the corresponding observed captures (1) and zero-encounters (0) of 6831 samples at 5169 trapping stations (right).**X-axis represents the coordinates of localities on each of the first (A) and second (B) principal components of the synthetic socio-spatial MCA In the right panel, the size of the dots is proportional to the total number of trapping stations and is expressed on a natural logarithmic scale.

Accounting for such effects on imperfect detection, the probability of occupancy of *R*. *rattus* varied with data of human geography (Table 3). The observed proportion of trapping stations occupied was 0.081 whereas accounting for imperfect detection suggests a ca. 1.8 times higher estimate of occupancy (0.148). The probability of occupancy of *R*. *rattus* decreased with the coordinates on the first principal component from the agricultural characteristics to the commercial characteristics, and decreased with the coordinates on the second principal component from a low degree of integration to the road, transportation, and commercial networks to a higher degree. On the second principal component the two gold mining villages appeared as exceptions since they were characterized by a low integration to the network but also show low probability of presence of *R*. *rattus* (Fig 3).

**Table 3:** Results from the occupancy models. *p* is the estimated probability of detection of *R*. *rattus*, *Ψ* is the estimated probability of occupancy of *R*. *rattus*. A dot indicates a constant parameter. Axis 1 and Axis 2 referred to coordinates on the two first principal components from the synthetic typology. Trap referred to the two models of traps, and Night to the two consecutive nights of trappings.

Model	AIC	deltaAIC	AIC wgt	Model Likelihood	no.Par.	-2*LogLike
Ψ(Axis 1, Axis 2), p(Night, Trap)	3102.77	0.00	0.9992	1	6	3090.77
Ψ(Axis 1, Axis 2), p(Trap)	3117.15	14.38	0.0008	0.0008	5	3107.15
Ψ(Axis 1), p(Night, Trap)	3126.17	23.40	0.0000	0.0000	5	3116.17
Ψ(Axis 1, Axis 2), p(.)	3140.86	38.09	0.0000	0.0000	4	3132.86
Ψ(Axis 2), p(Night, Trap)	3170.72	67.95	0.0000	0.0000	5	3160.72
Ψ(.), p(Night, Trap)	3205.29	102.52	0.0000	0.0000	4	3197.29
Ψ(.), p(Trap)	3217.22	114.45	0.0000	0.0000	3	3211.22
Ψ (.), p(.)	3239.64	136.87	0.0000	0.0000	2	3235.64

## Discussion

### Explaining Socio-Spatial Clusters through Historical and Contemporary Dynamics of Human Societies

The black rat distribution is strongly related to the socio-spatial clusters here defined. Thus, understanding the social processes involved in the construction by human societies of these spatial heterogeneities would allow the identification of the socio-spatial systems favorable to the invasion by the black rat. This section focuses on how these heterogeneities fall within socio-spatial systems built by inherited and current social dynamics.

Southeastern Senegal is a national periphery, an agro-pastoral space “reserve“, which has inherited from low overall human densities and a weakly structured settlement [35]. However the last two decades are characterized by a growing complexity of its human territory, thanks to the multiplication of nodal localities and the densification of exchange networks [36].

The synthetic socio-spatial typology that describes the degree of territorial structuring capacities and of integration among trade activities and transportation networks indeed confirm that the human geography of Southeastern Senegal cannot be reduced to the usual rural-urban categories [37], but covers a range of shaded situations illustrating these complex dynamics.

Urban-like localities (Goudiry, Kidira, Velingara) that form administrative, commercial, and transportation centers have originated from past colonial organization, mainly along railway stops established for the needs of groundnut trade [38,39]. Recently two phenomena have reinforced their capacities as commercial centers, connected towns and urban-like localities: the acquisition of chief town status through decentralization, and the soar of commercial activities on national road N°1 due to the increase of international trade towards Mali [40].

The combination of these administrative functions, economical activities, and spatial situations among Southeastern Senegal also plays a key role in defining rural localities with territorial structuring capacities (Kounkane, Kotiari, Bala, Sintiou Maleme, Kedougou, etc.). Again, the colonial legacy of groundnut trade [41–43] and the recent establishment of a decentralized land planning policy [37] have allowed the advent of these rural towns and their structuring territorial role. They are characterized by transportation line stations, commercial supplies (markets, numerous shops) and agricultural supplies such as agricultural cooperatives/warehouses of “Rural Communities”, collecting centers structuring the intervention areas of agro-industrial cotton exploitation.

Conversely, marginal rural localities (Dide Gassama, Boutougufara, etc.) do not benefit from these amenities. Because of long distance to roads and the lack of transportation facilities, they appear excluded from trade activities and act as self-subsiding villages (diversification strategies such as exploitation of natural resources, plus agriculture, own-storage management).

Eventually in the southeasternmost region of Kedougou, industrial and small-scale mining have come to challenge the traditional spatial organization [18,44] Gold mining villages, which no long ago were agricultural localities poorly interacting with the regional capital, are now real commercial centers (permanent markets, numerous shops) with daily links to Kedougou city. These renewed activities combined with a decentralized regional planning and a general willing of opening are likely to influence this territory. For instance, the completion project of the Transafrican road that connects Dakar to Bamako through Kedougou will contribute to reinforce these dynamics.

### Understanding Socio-Spatial Systems Favorable to the Spatial Diffusion of an Invasive Species

The unequal occurrence and abundance of the black rat can be partly considered as a result of social and spatial dynamics of human societies, in particular social and commercial links in this southeastern region of Senegal.

Previous studies have suggested the importance of structural and functional links in the black rat spread in Southeastern Senegal. Duplantier et al. [45] argued that under favorable ecological conditions the black rat distribution may only be limited by isolation. The latter then characterized the extreme south east of the country, from the Niokolo Koba National Park (NKNP) eastwards. They also noticed that its apparent absence at that time (in the 1980’s) in the eastern town of Bakel along the Senegal river (confirmed by a recent trapping session in April 2015), where its presence was documented by the end of the 19^th^ century [46], was likely related to the replacement of the fluvial trade way on the Senegal River by southern rail and road trades, through the central part of the country (see also [16]). Duplantier et al [47,41] predicted the invasion of the Kedougou area by the black rat as soon as the road through the NKNP will be asphalted and this was confirmed two years later [48]. In a context of dead-end near a “cold border” [49] and thanks to an increase of perennial flows allowed by the asphalting, the regional capital of Kedougou was the first place reached by the rodent. Population genetics analysis [16] demonstrated that the black rat was certainly directly propagated via human-mediated long-distance dispersal from Tambacounda and this particular type of diffusion, ie hierarchical diffusion, fits well with the absence of settlement continuity along the road, intersected by the NKNP, the lack of relay town and the absence of the black rat in all localities along the road between the NKNP and Kedougou town [50].

Still now, the structuration and organization of the human geography of Southeastern Senegal contribute to explain the rodent situation among our socio-spatial clusters.

- In the southeastern Kedougou region, the main way of rodents’ introduction remains national road N°7 through the regional chief town. The soar of trade activity in gold mining villages has effectively led to increased trade and circulations in this area but almost exclusively directed toward Kedougou, the regional capital and the only significant supply place for the region. Indeed, the transportation lines able to supply traders all start from Kedougou, whereas lines from Tambacounda do not reach the southeasternmost gold perimeters. Although these gold mining villages may be the most structurally vulnerable to future colonization by *R*. *rattus* in terms of introduction opportunities, it is probable that gold mining implies transportation flows for the limited period of gold exploitation and through fractioned carriage, which may not be suitable for rodent successful (or long term) establishment. Moreover, Mbodj [44] has stated that spatial changes in the mining context are still embryonic in the region. In this context, we hypothesize that the future opening of an effective custom office on the south Transafrican road [51] will challenge the diffusion potentialities in the extreme south east by securing perennial and significant flows, enabling *R*. *rattus* to sustainably colonize new localities in this area.
- In the areas north of NKNP and along tarred roads (national roads N°1, N°6, N°7 Fig 1), *R*. *rattus* is well installed. There, Konečný et al. [16] have shown that the rodent has probably spread along N°1 road from the regional capital Tambacounda. Moreover, its eastward expansion since the 1990’s is likely induced by the same phenomena of road asphalting and traffic increase that took place more recently across NKNP.
- We could have thought that northern rural margins outside national road N°1 would not have been suitable for the black rat diffusion because of rural tracks likely to reduce flows. On the contrary, our results suggest that these “marginal rural localities” are highly favorable to the rodent establishment. This can be understood by examining their vocations and functioning. In the northwestern part of Tambacounda region, the “useful” Tambacounda hinterland directly linked to the regional capital is characterized by cotton cash crops, charcoal and other agricultural activities [36,52]. Therefore, even as margins, these human territories benefit from regular exchange flows of agricultural products representing as many occasions of rodent introduction. This is the case of the Goumbayel / Dianke Makha / Soutouta area situated along a main axis (Fig 1) with potential higher traffic oriented towards Tambacounda.
- By contrast, isolated rural localities with a self-subsiding tendency are characterized by a weak integration limiting diffusion potentialities (Dide Gassama/Doulouyabe area or Ndiobene). But here again, functioning challenges structures. Indeed as long as the logic of charter prevails [49], even the least integrated margins could be submitted to flows. For instance at Dide Gassama, a small isolated agricultural village, an extended notable family generates important flows from main towns to ensure the supply of their hundreds of residents. Without any other significant traffic, this phenomenon is probably involved in the (potentially recurrent) immigration episodes of the black rat, found to be the only rodent species captured in this village at the time of our survey.

### Socio-spatial Systems: Necessary but not Sufficient Conditions to Understand the Invasive Processes and Patterns

Combining structural links and functional flows among socio-spatial systems however does not permit to fully understand the invasion of the black rats. Indeed, along the northern tarred road (N°1), one could imagine *R*. *rattus* to be well installed in major urban towns thanks to the important (and increasing) flows in volume and in frequency due to international trade [40]. Conversely, our results suggest that major and integrated nodes, such as urban-like localities (Bala, Goudiry, Kidira) are not especially favorable to the rodent. Important flows along national road N°1 may contribute to feed the rodent populations by regular immigrants but other parameters need to be considered to explain why the species does not reach higher relative abundance there. In particular because the invasive process also implies phases of establishment and proliferation, the many conditions of the species habitat suitability should be considered.

- Local ecological conditions may be determinant in the success of the species. These environmental conditions include i) the animal community, for instance other small mammal species, including the invasive house mouse (*Mus musculus*) that has recently expanded in urban-like localities along national road N°1 [53], or large shrews (*Crocidura olivieri*) as potential competitors, and carnivores a predators; ii) building materials: the use of cement and corrugated iron has been expanding, in place of more traditional materials, as adobe and thatched roof, that may have been much more favorable to black rats.Furthermore, the region of Kedougou is the main area of another true and native commensal rodent, *Mastomys natalensis*, which is a main competitor of the black rat. There the black rat is not yet established. Above the human geography of this region, such as limited connectivity to networks, the presence of this rodent in gold mining villages may act as a limiting factor for the black rat establishment [54].
- A non-exclusive explanation may lie in the differential use of harvest storage facilities that provide potentially abundant and sustainable food to rodents. In this field, marginal rural localities often store important amounts of foodstuff because of the difficulty to reach supply places during the rainy season. By contrast, commercial towns may favor daily consumption, particularly non-agricultural households, enhanced by easy access to markets and shops, resulting in reduced foodstuff availability for the rodents at both the locality and household level.

## Conclusion

In a region of recent and ongoing establishment of a major invasive species, *R*. *rattus*, we have demonstrated that urban-like localities but also localities with territorial structuring capacities and rural margins could differentially act as socio-territorial meshes to favor the rodent spread. Our study suggests that the spatial diffusion of this invasive rodent species could be driven both by *(i)* spatial structural characteristics (e.g. roads and transportation networks), and *(ii)* the actual functioning of the human territory, ie social and spatial interactions. These two socio-spatial drives result from both inherited and current entangled dynamics of the human societies and their territory.

Identifying the functional dimensions of diffusion is a major prerequisite to distinguish real from potential diffusion area. Local ecological conditions certainly influence the invasive process, eventually acting as limiting factors, but the current opening dynamics of the Southeastern Senegal undoubtedly modulates the probabilities of rodent introduction, establishment, and proliferation. Thus the spatial and social structure and functioning of human territories, represent essential aspects for addressing potential risks of zoonosis diffusion.
